# Retention properties and mechanism of agricultural waste maize whisker on atmospheric mercury

**DOI:** 10.1186/s40643-023-00683-y

**Published:** 2023-09-29

**Authors:** Guiling Zheng, Qianxiu Chen, Feng Zhou, Peng Li

**Affiliations:** 1 School of Resources and Environment, Qingdao Agricultural University, Qingdao, 266109 Shandong China; 2 School of Food Science, Nanjing Xiaozhuang University, Nanjing, 211171 Jiangsu China

**Keywords:** Air pollution, Biosorption, Heavy metal, Porous material

## Abstract

Mercury (Hg) is a global pollutant transmitted mainly through the atmosphere, posing a serious threat to biological survival and human health. Porous materials, with high specific surface area, high porosity, and high adsorption, are particularly suitable for the purification of atmospheric Hg mixtures. However, plant porous materials are rarely directly used for atmospheric Hg purification. In this study, the properties and mechanism of maize whisker in removing atmospheric Hg were analyzed. The results show that the Hg content in the whiskers increases significantly as the initial Hg concentration increases, and 79.38% Hg can be removed by 0.2 g maize whiskers after 1 h exposure when the initial Hg concentration is 0.1 μg m^−3^, indicating that maize whiskers can accumulate atmospheric Hg rapidly and effectively. The hole diameter of the maize whisker is between 0.83 and 3.06 μm, which is suitable for the adsorption of small substances. Correlation analysis shows that maize whiskers have a significant correlation between atmospheric Hg retention and its specific surface area, pore size, medium pore ratio, and micropore ratio, suggesting that the maize whisker hole feature has a significant influence on its ability to retain atmospheric Hg. Compared with the energy profiles before and after Hg treatment, the peak of Mg decreased after Hg adsorption. Fourier infrared spectrometer analysis suggests that functional groups such as -OH, -COOH, and -O- are involved in the adsorption process. The change in pH value shows an obvious effect on the overall change in zeta potential in the adsorption process. Therefore, a variety of mechanisms, including physical adsorption, electrostatic adsorption, complexation, chelation, and ion exchange, are involved in Hg retention with the maize whisker. This study reveals the important potential value of agricultural waste maize whiskers in the purification of atmospheric heavy metal Hg.

**Graphical Abstract:**

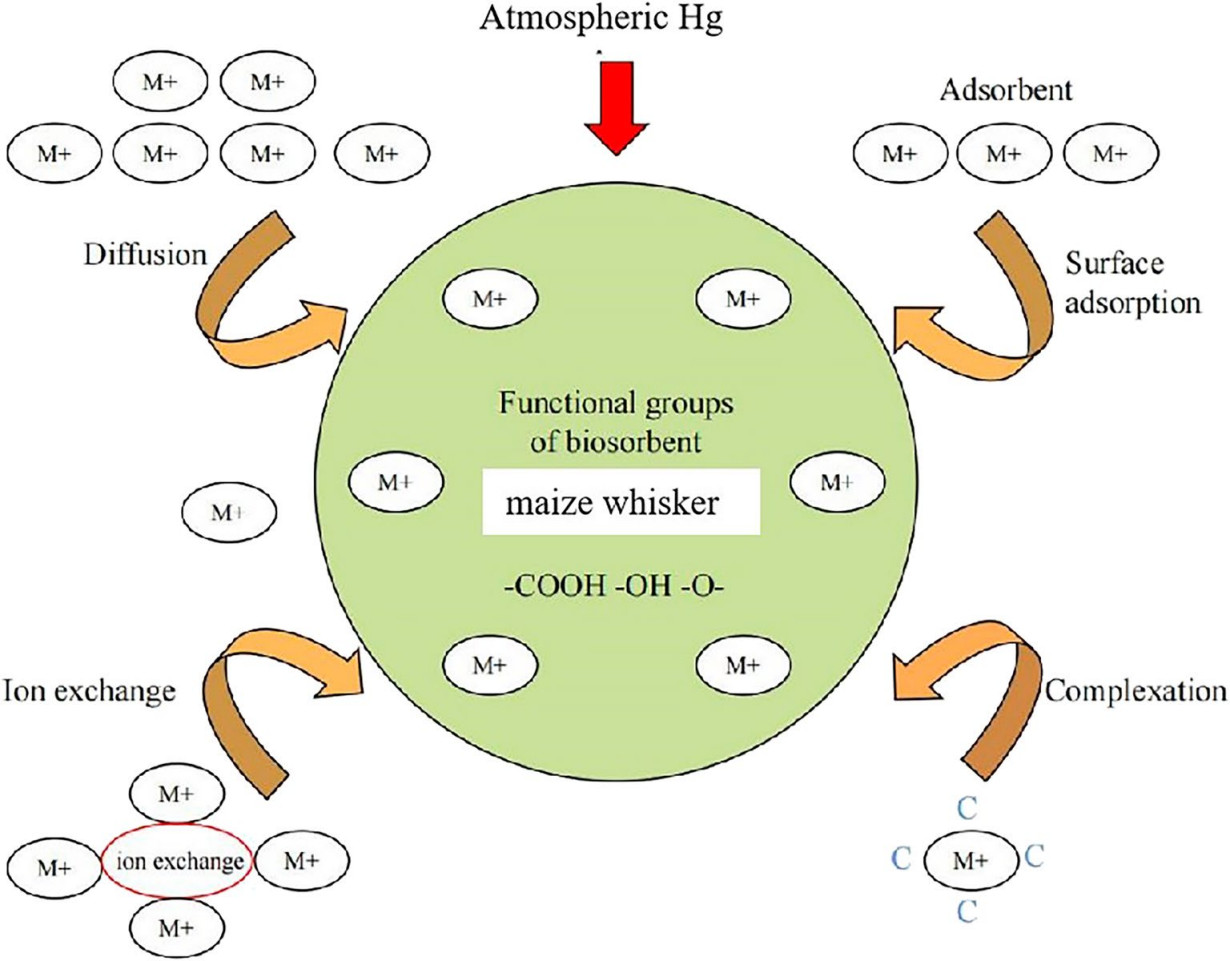

**Supplementary Information:**

The online version contains supplementary material available at 10.1186/s40643-023-00683-y.

## Introduction

Mercury (Hg) is a global pollutant transmitted mainly through the atmosphere and is present even in the Arctic region (Obrist et al. 2017). It is highly toxic and bioenriched, posing a serious threat to biological survival and human health (Natasha et al. 2020). Hg sources in the atmospheric environment are complex, including natural sources (volcanic and geothermal activities, forest fires) and human sources (fossil fuel combustion, nonferrous metal smelting, cement manufacturing, waste incineration) (Pavithra et al. 2023). The Hg form is also complex and diverse, including atomic Hg (Hg^0^), gaseous Hg oxide (Hg^2+^), and granular Hg (HgP) (Tsui et al. 2020), which improves the difficulty of Hg removal. At present, there are two main methods of Hg removal. First, the existing wet desulfurization device can remove 80–95% Hg^2+^ in flue gas, but the capture effect of Hg^0^ is not significant (Pavlish et al. 2003). The second is to remove the Hg in the flue gas through the adsorption of activated carbon and other adsorbents, which is also the main postcombustion Hg removal technology (Chai et al. 2022). Therefore, the selection or manufacture of an effective adsorbent is of great significance for the treatment of atmospheric Hg pollution.

Porous materials, with high specific surface area, high porosity and high adsorption, can separate one or more substances in mixtures and are theoretically particularly suitable for the purification of atmospheric Hg mixtures (Ma et al. 2018; Wang et al. 2020). At the same time, after hundreds of millions of years of natural selection, the plant microstructure structure presents a natural hierarchical structure, with multilevel, multiscale tubular, cellular or fibrous structural morphological features and micro- and nanosized structures (Wegst et al. 2015). The specificity, widespread existence, and renewability of plant microstructures after natural selection have inspired researchers to develop novel porous materials (Zanoletti et al. 2020). Many natural plant byproducts, such as corn stalks, peanut shells, orange peel, tea leaves, and pine sawdust, have been directly taken or modified into good adsorbents for metal ions and organic matter in wastewater (Ngah et al. 2008; Fomina and Gadd 2014). However, the main commonly used Hg adsorbents are activated carbon, fly ash, calcium-based adsorbents, and ore adsorbents (Kogut et al. 2021; Pavithra et al. 2023), but plant porous materials are rarely directly used for atmospheric Hg purification.

Maize whisker is the female inflorescence (including style and stigma) of maize (*Zea mays* L.) and is one of the main byproducts of maize harvest (Hasanudin et al. 2012). In recent years, some scientists have tried to use maize whiskers to absorb pollutants in wastewater. The results show that it can effectively adsorb heavy metal ions such as lead, copper, zinc, cobalt, nickel, and oil substances in wastewater (Bondada et al. 2004; Zhu et al. 2013; Yu et al. 2016; Sabri et al. 2019; Lakshmi et al. 2021). The holes inside the maize whisker are considered to enhance the adsorption of the smaller size matter (Asadpour et al. 2015; Petrovic et al. 2016, 2017). Our study also showed that maize whisker can effectively retain atmospheric fine particles of PM_2.5_ for a long period of time (Zheng et al. 2019). However, whether maize whisker can effectively retain Hg in the atmosphere and its corresponding mechanism has not been reported. Therefore, in this study, the properties and mechanism of maize whisker in removing atmospheric Hg were analyzed, in order to facilitate the application of agricultural waste for the removal of atmospheric heavy metals.

## Materials and methods

### Materials

Maize whisker is harvested from the variety of corn Denghai 605 in the farmland of Qingdao. Referring to the method of Petrovic et al. (2017), the maize whisker was cleared with deionized water, put into an oven, and dried at 80 ℃. The whisker powder was obtained after the maize whisker was crushed by a high-speed grinder. Four kinds of powder with different particle sizes, i.e., 75 μm, 100 μm, 150 μm, and 270 μm, were obtained after the powder was passed through sifters of different sizes.

### Treatment of maize whiskers with atmospheric Hg

**Fig. 1:**
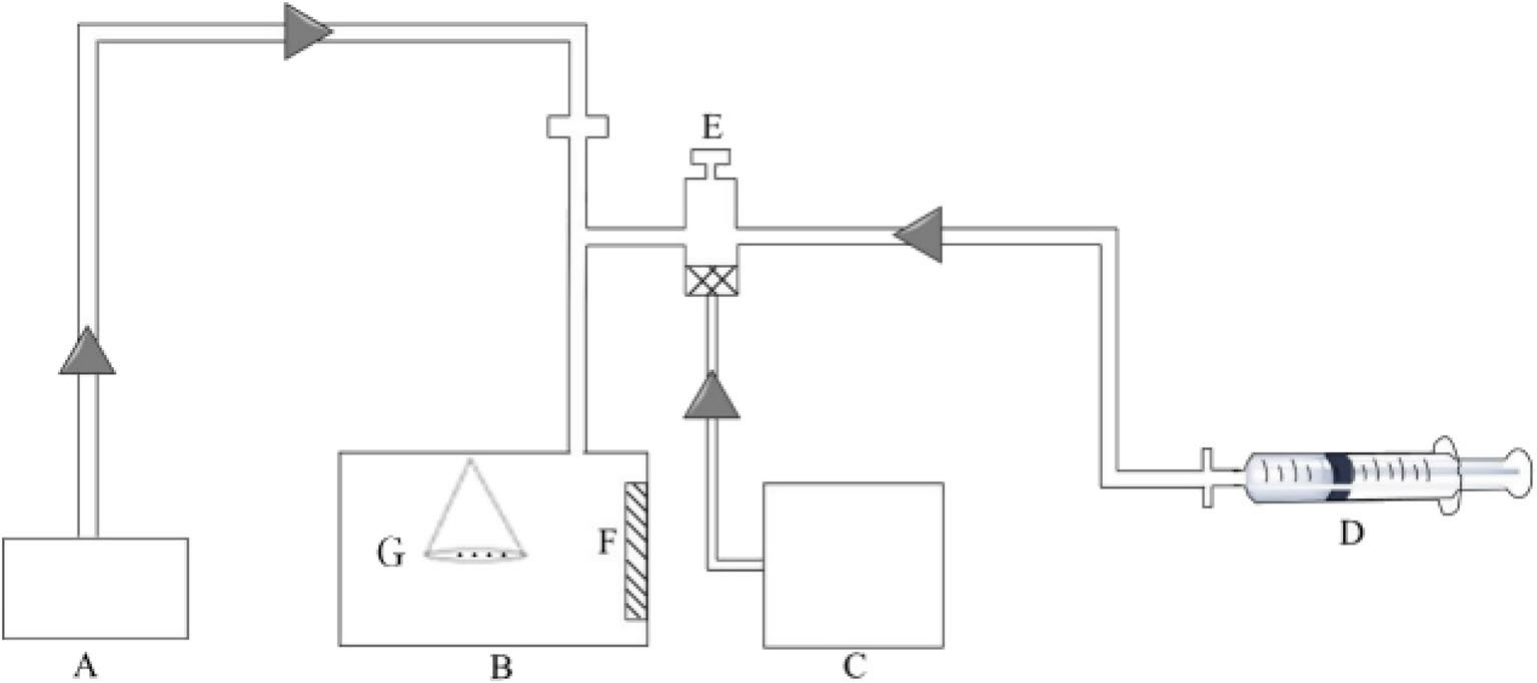
Schematic diagram of atomization exposure system. **A**, Air pump; **B**, Exposure chamber; **C**, Compressor; **D**, Syringe; **E**, Atomizing cup; **F**, Fan; **G**, 400 mesh sifter with maize whisker powder in it

The experiments were performed in an aerosol exposure box (Fig. 1B) with polymethyl methacrylate (PMMA) material of 0.1 m^3^. The box is connected to the air pump (Fig. 1A), the compressor (Fig. 1C), and the atomizer (Fig. 1E), which provides continuous air source power for the atomization system. The atomizer atomizes the Hg standard solution in the external syringe (Fig. 1D) to create an aerosol atomization environment. A fan (Fig. 1F) in the box rotated at a uniform speed to make the Hg mix evenly and quickly. To reduce the impact of the injection time, the volume of Hg solution was uniformly set to 30 mL, and the syringe injection speed was set to 15 mL h^−1^. The amount of Hg solution is calculated according to the set initial concentration of Hg and the volume of the atomization box. The prepared maize whisker powder was spread in a 400 mesh sifter (Fig. 1G) and suspended in the box for atomization treatment.

### Determination of Hg removal efficiency for maize whisker

The retention of Hg (μg g^−1^) was calculated according to the following formula:

$$Rf = Ct*{{\text{V}} \mathord{\left/ {\vphantom {{\text{V}} {{\text{Ws}}}}} \right. \kern-0pt} {{\text{Ws}}}}$$

where Ct (μg L^−1^) is the concentration of Hg in the digestion sample after the retention time *t*, V (*L*) is the volume, and Ws (*g*) is the mass of the maize whisker.

The removal efficiency (%) of Hg was calculated by the following formula:

$${\text{P}} = {\text{ }}\left( {{\text{Rf }} - {\text{ Ri}}} \right)*{\text{Ws}}/{\text{W}}_{{{\text{Hg}}}} *{\text{1}}00\%$$

Ri (μg g^−1^) is the background value of Hg in maize whisker, and W_Hg_ (*g*) is the content of Hg added to the exposure box, which can be converted from the volume of the exposure box and the volume of the standard Hg solution added.

Determination of Ct value:

After Hg treatment, a certain mass of maize whisker was placed in a 25 ml colorimetric tube and digested with a 4:1 mixture of concentrated nitric acid and perchloric acid. Three blank solutions were digested following the same procedure Three blank solutions were digested following the same procedure as the quality control of Hg measurement. The concentration of Hg (μg L^−1^) in each sample was determined by an atomic fluorometer (AFS-933, Jitian Instruments, Beijing, China).

### Effect of different factors on Hg retention

The experiment was conducted with a whisker powder size of 150 μm. Five gradients were set for maize whisker mass, exposure time, initial Hg concentration, and pH value. Hg exposure experiments were performed according to the combination of conditions in Table 1.

**Table 1:** Factors influencing Hg retention with maize whiskers

Experimental objective	Mass of maize whiskers (*g*)	Exposure time (*h*)	Hg initial concentration (μg m^−3^)	pH value
Effect of mass of maize whiskers	0/0.1/0.2/0.3/0.4	2	0.1	4
Effect of exposure time	0.2	0/1/2/3/4	0.1	4
Effect of Hg initial concentration	0.2	2	0/0.05/0.1/0.2/0.4	4
Effect of pH value	0.2	2	0.1	2/3/4/5/6

### Characteristics of maize whisker before and after Hg treatment

In this part of the experiment, a maize whisker size of 150 μm was also used as the material. The Hg stress conditions included 0.1 g of maize whisker, an initial concentration of maize of 0.1 μg m^−3^, an initial pH value of the Hg solution of 4, and an exposure time of 2 h.

#### Surface and cross-section structure

The surface and cross-sectional structures of maize whiskers were observed using a scanning electron microscope (Nova nano 450, FEI, USA). A sample of dried maize whisker powder was gently applied to the conductive adhesive, blown out with an ear suction ball to distribute it evenly, and then placed into the gold plating device with carbon spraying and gold plating. The acceleration of the electron gun was 10.0 kV, and it was scanned and photographed from whole to local at the appropriate magnification.

#### Energy spectrum

The surface and cross-section elements were analyzed using an energy spectral dispersion X-ray spectrometer (Nova nano 450, FEI, USA). In the SEM field of view, 5 points were randomly selected for point scanning for energy spectrum analysis.

#### Functional groups

The spectra before and after Hg treatment were compared by a Fourier infrared spectrometer (FTIR, Nicolet iS5, Thermo). The dried potassium bromide and the sample were mixed at a mass ratio of 50:1 to 100:1, and the infrared spectrum of the sample was obtained in the spectral range of 4000 to 400 cm^−1^ with a resolution of 4 cm^−1^ and 32 scans.

#### Zeta potential

The zeta potential was analyzed using a zeta potentiometer (Zetasizer Nano ZS ZEN3600, Malvern, UK). Maize whisker powder (0.4 g) was placed in a 250 mL conical flask, and 150 mL of distilled water was added. Then, the pH was adjusted with 0.1 mol of L^−1^ NaOH (1.0, 2.0, 3.0, 4.0, 5.0, 6.0). The mixture stood at room temperature for 17 h before taking the supernatant to measure the zeta potential of maize whisker powder.

### Effect of maize whisker hole features on Hg retention

#### Characterization of maize whisker hole structure

The specific surface area, pore volume, and pore size distribution of maize whisker samples were determined by an automatic specific surface area and pore analyzer (Micromeritics, ASAP 2460, USA). The samples were dehydrated for 2 h at 105 ℃ under vacuum before adsorption. The adsorption‒desorption isotherm of maize whisker was determined by high purity *N*_2_ using the ASAP system at liquid nitrogen temperature. The total surface area of the sample is calculated by the BET method, the medium pore surface area is calculated by the t-plot method, and the micropore surface area is the difference minus the total surface area. The total hole volume is regarded as the volume of the liquid nitrogen absorbed when the relative pressure is 0.95. The sample volume is calculated by the t-plot method. The medium volume is the difference minus the total hole volume. The mean aperture and micropore aperture of the sample are calculated by the BJH method.

#### Analysis of Hg retention performance of maize whiskers with different sizes

Hg retention of maize whisker was tested using four maize whiskers (75 μm, 106 μm, 150 μm, and 270 μm) with 0.1 g of maize whisker, an initial concentration of 0.1 μg m^−3^, a concentration of 4 at the initial pH of the Hg solution, and an exposure time of 2 h.

### Data analysis

Statistical analysis of the obtained data was performed using IBM SPSS 23.0, and the mapping work was performed using Origin Pro 9.0. If the data met the normal distribution and homogeneity of variance test, the difference between Hg content under different conditions was analyzed by one-way ANOVA (one-way analysis of variance), and the correlation between structural parameters of maize whisker hole and Hg retention was analyzed, expressed as Spearman's correlation coefficient. If the data did not fit, the Kruskal‒Wallis test was used to determine whether the differences between the indicators were significant, and *p* < 0.05 was considered significant.

## Results

### Influence factors of atmospheric Hg retention with maize whiskers

#### Effect of maize whisker mass on Hg retention performance

**Fig. 2:**
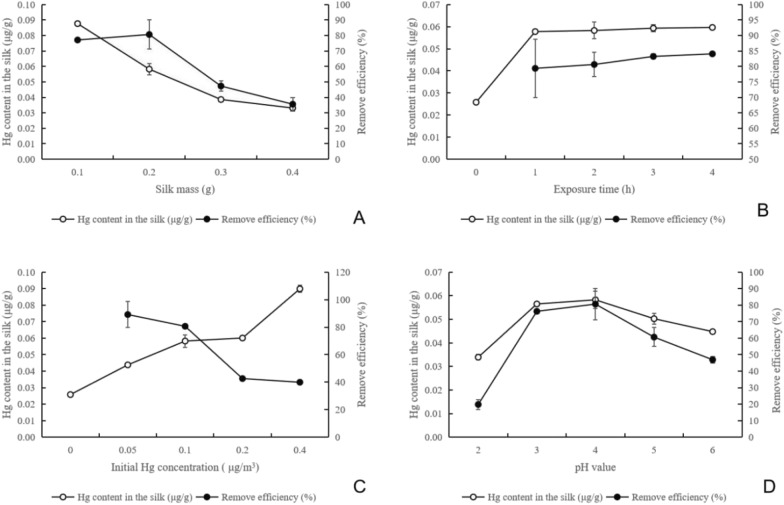
Factors influencing Hg retention with maize whisker **A**, Effect of maize whisker mass; **B**, Effect of exposure time of Hg; **C**, Effect of initial Hg concentration; **D**, Effect of Hg pH value

With the increase in maize whisker mass, the retention of Hg in maize whiskers gradually decreased (Fig. 2A), from the highest value of 0.088 μg g^−1^ to 0.033 μg g^−1^, with significant differences between treatments (*p* < 0.05). However, the Hg removal efficiency of maize whiskers with different masses did not change linearly (Fig. 2A). When the mass of maize whiskers was 0.1 g, the removal rate of Hg was 77.08 ± 0.79%. When the corn maize whisker amount increased to 0.2 g, the Hg removal rate increased to 80.63 ± 9.39%. Then, with increasing maize whisker amount, the Hg removal rate decreased significantly (*p* < 0.05).

#### Effect of exposure time on Hg retention

With prolonged exposure time, the retention and removal efficiency of maize whiskers showed an increasing trend (Fig. 2B). In the first 1 h, the retention of maize whiskers increased rapidly (*p* < 0.05), reaching 0.0578 ± 0.0008 μg g^−1^, and the removal rate reached 79.38 ± 1.65%. With the extension of time, the retention amount and the removal rate increased slowly, and there was no significant difference from 1 to 4 h (*p* > 0.05).

#### Effect of the Hg initial concentration on Hg retention

With the increase in the initial concentration of Hg, the retention of Hg in maize whiskers increased significantly (*p* < 0.05), but the removal rate gradually decreased (*p* < 0.05) (Fig. 2C). The maximum removal rate was 89.17 ± 2.60% at an initial Hg concentration of 0.05 μg m^−3^.

#### Effect of pH on Hg retention

With increasing pH, the retention of atmospheric Hg tended to increase first and then decrease (Fig. 2D). At pH 4, both the Hg retention and removal rates were 0.0583 ± 0.0038 μg g^−1^ and 80.63 ± 9.39%, respectively, which were significantly higher than those at pH 2 and 3 (*p* < 0.05). After the increase in pH, the removal rate and retention decreased, but there was no significant difference from the results at pH 4 (*p *> 0.05).

### Characteristic changes before and after Hg treatment

#### Surface and cross-section structure

**Fig. 3:**
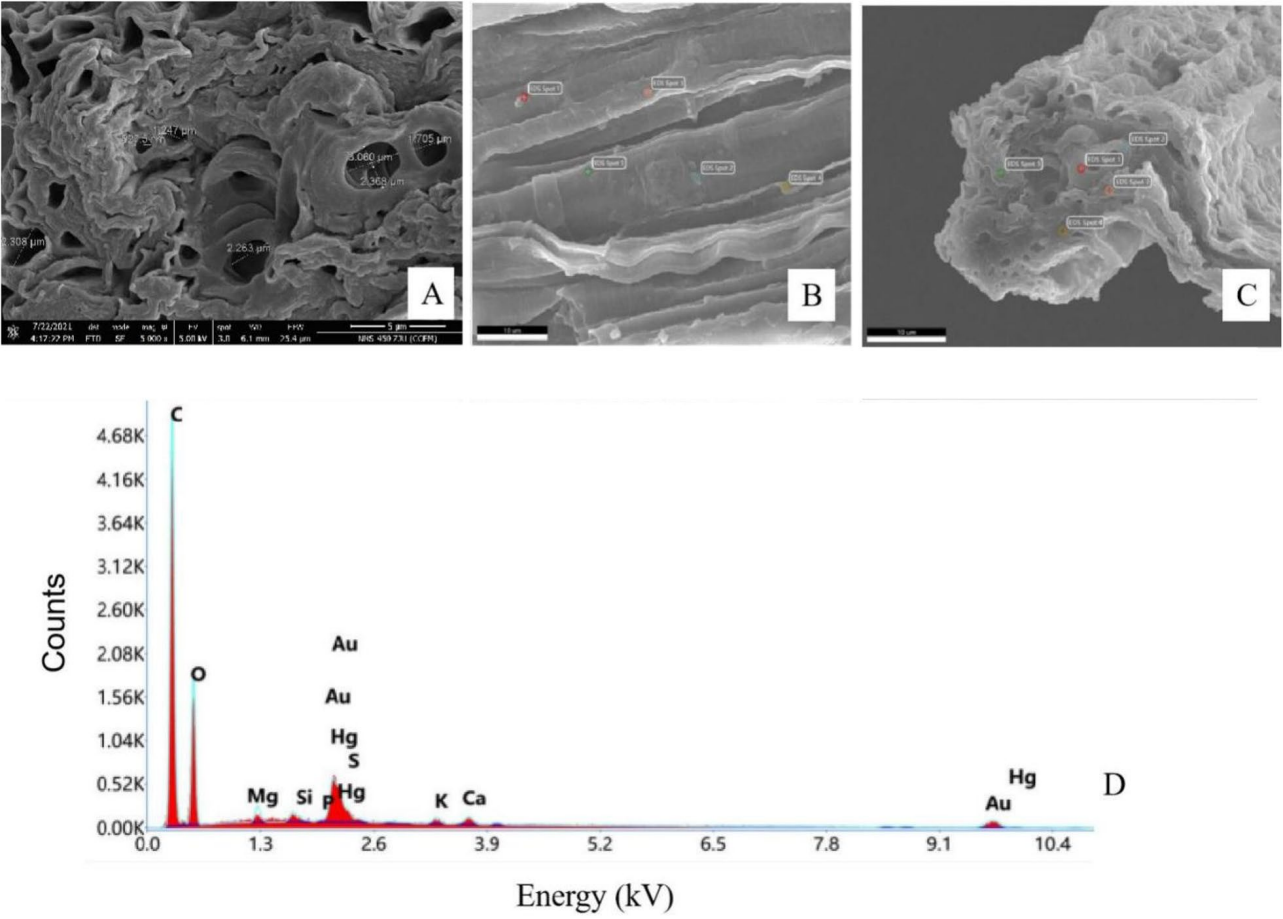
SEM structure and energy spectrum analysis of maize whiskers. **A**, cross section of maize whiskers; **B**, surface of maize whiskers; **C**, scanning sites on the cross section of maize whiskers; **D**, energy spectrum after Hg treatment

The surface of maize whiskers is rough with wall folds (Fig. 3B), and there is a hole structure inside. Those with a regular shape are the catheter, while irregular is the tissue gap. The diameter of the hole was between 0.83 and 3.06 μm (Fig. 3A). There were no significant changes in the surface and section structure of the maize whiskers after Hg treatment.

#### Analysis of the energy spectrum

EDS analysis (Fig. 3B, C) shows that essential elements such as C, O, P, and S and trace elements such as K, Si, Mg, and Ca can be detected in maize whiskers, in which C and O have the highest percentage of weight. Hg was not detected inside the untreated Hg-stressed maize whiskers, while Hg was detected in the 0.05 μg m^−3^ Hg-treated whiskers (Fig. 3D). The relative contents of calcium, magnesium, and potassium were high before Hg treatment, while only magnesium decreased after Hg treatment (Additionl file 1: Table S1).

#### IR spectrum

**Fig. 4:**
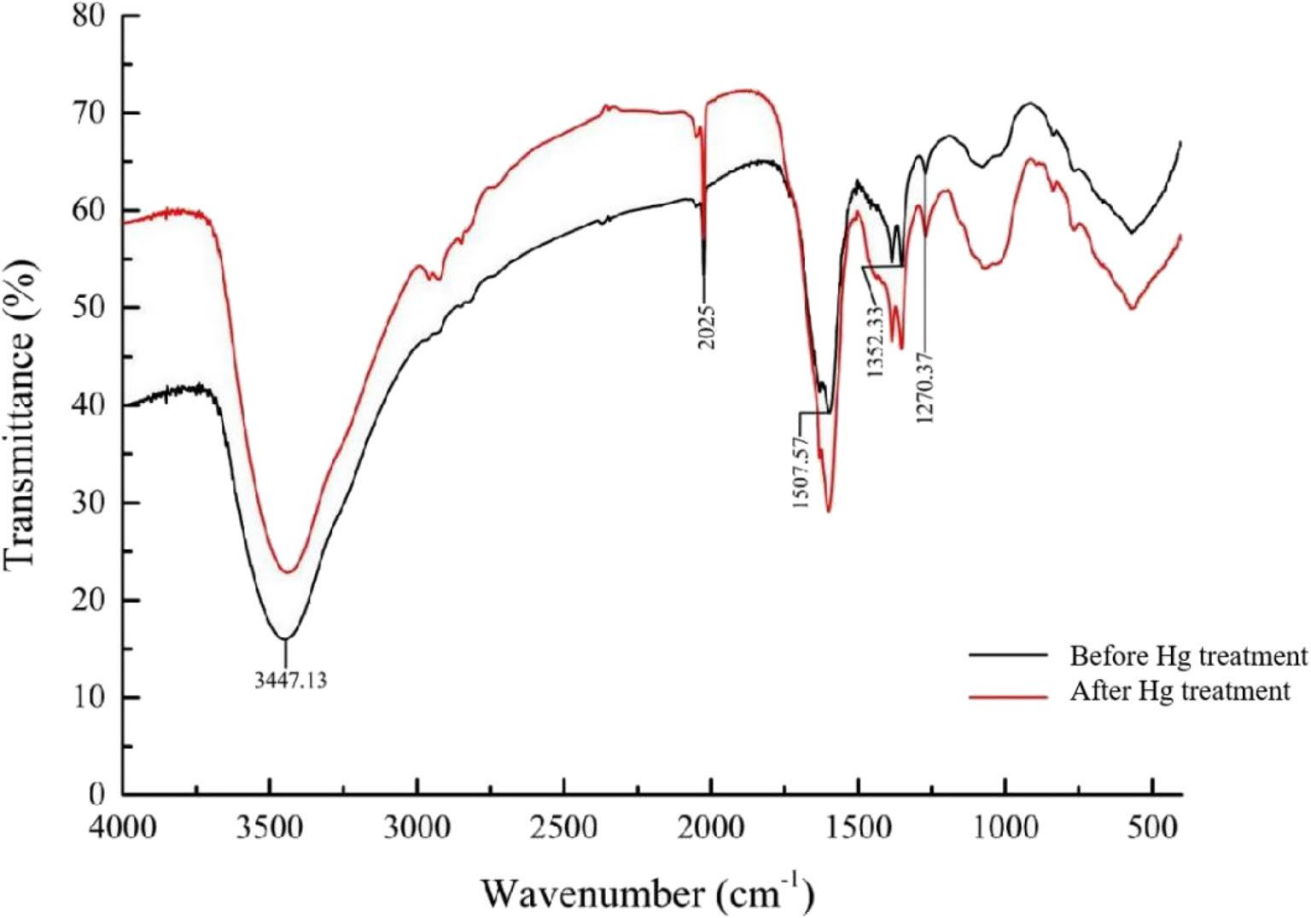
The FTIR spectrum of maize whiskers before and after Hg treatment

The FITR profile (Fig. 4) shows the changes in the intensity and location of the six absorption peaks after Hg treatment (Additionl file 1: Table S2). The peak at 3447.13 cm^−1^ is the characteristic peak of absorption of the intermolecular H bond O–H stretching vibration in the polysome, with the peak increasing from 3447.13 to 3439.42 cm^−1^ with a significant decrease in intensity. The absorption peaks at 1507.57 cm^−1^, 157 cm^−1^, and 1352.33 cm^−1^ move toward high wavenumbers, and the absorption peaks at 3447.13 cm^−1^, 2025.85 cm^−1^, 1384.63 cm^−1^, and 1270.86 cm^−1^ move toward low wavenumbers, with the largest offset of the -OH absorption peak and the displacement of 8 cm^−1^.

#### Zeta potential

**Fig. 5:**
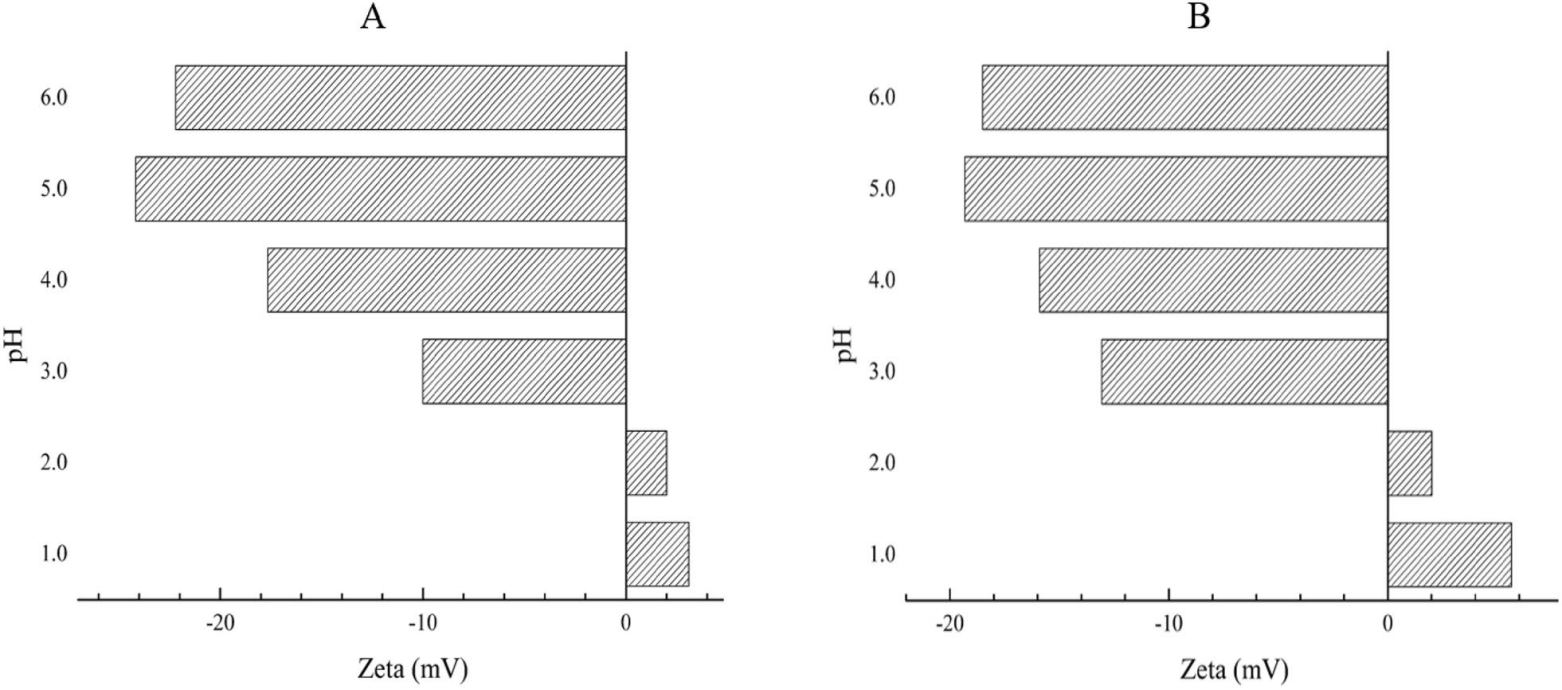
The zeta potential of maize whiskers before and after Hg treatment. **A**, before Hg treatment; **B**. after Hg treatment

When the pH value increased from 1 to 6, the zeta potential before Hg retention ranged from 3.11 to − 22.20 mV to 25.31 mV, and the zeta potential after Hg retention ranged from 5.65 to − 18.50 mV with a change of 24.15 mV (Fig. 5). At pH = 1.0 and pH = 2.0, when the pH ranges from 3 to 6, with increasing pH, the negative charge first increases and then decreases, reaching a maximum at pH = 5.

### Effect of maize whisker hole characteristics on Hg retention

#### Characteristics of maize whisker holes with different sizes

##### Specific surface area

With the continuous decrease in maize whisker size, the specific surface area, microhole surface area, and midhole surface area all increase accordingly (Table 2). The specific surface area of the smallest size is 1.5977 m^2^ g^−1^, and the specific surface area of the largest size is 0.2708 m^2^ g^−1^, which is approximately 5.9 times that of the latter. In addition, the medium pore surface area of four different size whiskers was higher than the micropore surface area.

**Table 2:** Pore structure characters of maize whiskers with different diameters

Diameter of maize whiskers (μm)	75	100	150	270
Total specific surface area (m^2^ g^−1^)	1.5977	1.3061	0.4723	0.2708
Micropore-specific surface area (m^2^ g^−1^)	0.204	0.187	0.0863	0.0649
Medium hole-specific surface area (m^2^ g^−1^)	1.3937	1.1191	0.386	0.2059
Total hole volume (cm^3^ g^−1^)	0.000240	0.000269	0.000373	0.000398
Micropore volume (cm^3^ g^−1^)	0.000044	0.000043	0.000037	0.000033
Medium hole volume (cm^3^ g^−1^)	0.000196	0.000226	0.000336	0.000365
Aperture (nm)	2.0669	2.4728	2.6545	3.9686

##### Hole volume

With increasing corn whisker particle size, the micropore volume decreased accordingly, but the total pore volume and the medium pore volume increased accordingly (Table 2). Compared with the micropore volume, the medium pore volume of different grain sizes is larger, accounting for more than 80% of the total pore volume.

##### Aperture distribution

The pore size of corn whiskers includes micropores and medium holes, and the distribution is concentrated within 50 nm, mainly between 10 and 20 nm (Table 2). The comparison of the four sizes of whiskers showed that as the whiskers’ size decreased, the corresponding midhole distribution decreased, and the mean pore size decreased accordingly.

##### Adsorption‒desorption isothermal curve

The adsorption‒desorption isotherm of maize whiskers of each size is similar (Additional file 1: Fig. S1), which is a typical multimolecular adsorption process. When the relative pressure (*P*/*P*_0_) is in the low-pressure Section (0.0–0.1), the gas adsorption capacity increases rapidly. With the increase in the relative pressure (*P*/*P*_0_), the microhole filling ends, that is, the first layer of adsorption has reached the saturation degree. At this time, the isothermal adsorption curve will exhibit a relatively clear turning point, and then multimolecular layer adsorption will begin. At the medium pressure Section (0.3–0.8), as the number of adsorption layers increases, the adsorption amount also gradually increases. When the relative pressure (*P*/*P*_0_) reaches the high-pressure Section (0.9–1.0), the adsorption curve increases sharply. It is worth noting that when the relative pressure is (*P*/*P*_0_) at 0–0.9, there is a lag ring between the adsorption‒desorption curves, and the area of the whisker particle decreases.

#### Effect of maize beard hole structure on Hg retention

##### Hg retention effect of maize whiskers with different particle sizes

**Fig. 6:**
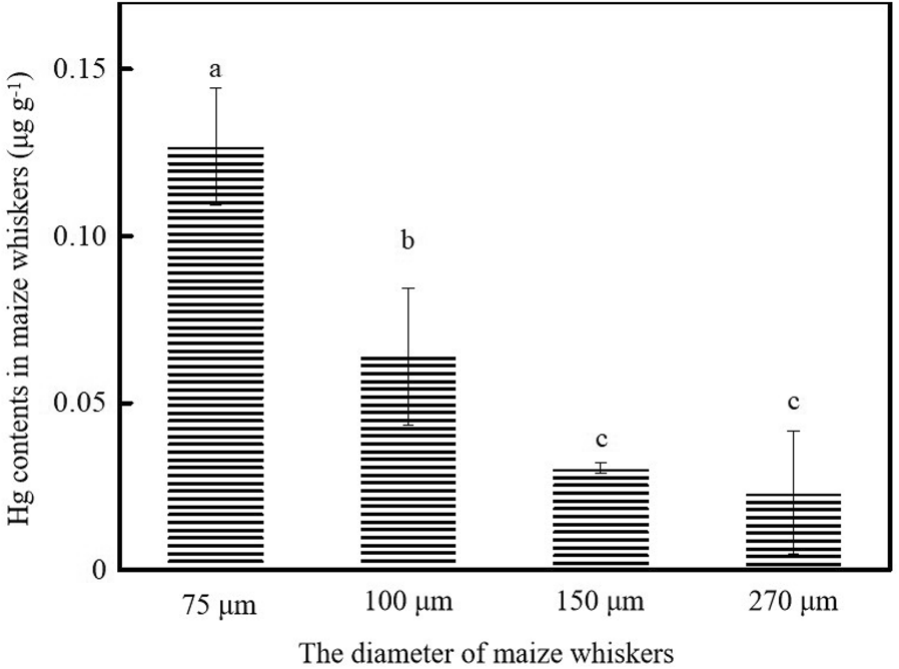
Effect of the diameter of maize whiskers on Hg retention

The retention of Hg in maize whiskers gradually decreased with increasing whisker size (Fig. 6). The maize whiskers with the smallest particle size retained the most atmospheric Hg, reaching 0.13 ± 0.02 μg g^−1^, which was significantly higher than the other three maize whiskers (*p* < 0.05).

##### Correlation analysis of Hg retention and structural parameters of maize whiskers

Correlation analysis shows that maize whiskers have a significant correlation between atmospheric Hg retention and its specific surface area, pore size, medium pore ratio, and micropore ratio. Among them, the surface area (Pearson correlation coefficient = 0.879, *p* < 0.01), micropore ratio (Pearson correlation coefficient = 0.884, *p* < 0.01), and significant negative correlation with pore volume, pore size, and pore ratio (Pearson correlation coefficient = − 0.861, − 0.725, − 0.884, *p* < 0.01).

## Discussion

The results of Hg retention in maize whiskers show that the Hg content in the whiskers increases as the initial Hg concentration increases (Fig. 2C), and this accumulation process can be completed with few maize whiskers (Fig. 2A) and a very short time (Fig. 2B), indicating that maize whiskers can accumulate atmospheric Hg rapidly and effectively. Hg is a global pollutant mainly transmitted through the atmosphere, so as a structure in direct contact with the atmosphere, the plant surface has a more direct and strong impact on Hg absorption and release. The exchange process between the plant surface and atmospheric Hg is the basic form of its participation in atmospheric Hg expenditure (Zhou et al. 2021). Many studies have shown that plants absorb atmospheric Hg^0^ through leaf stomata (Ericksen et al. 2003; Meng et al. 2018) and dry and wet settled atmospheric Hg^0^, Hg^2+^, and granular Hg through the plant surface (Rea et al. 2001; Wang et al. 2021). Our recent study also showed that maize whiskers can effectively retain fine particles for a long time (Zheng et al. 2019), and heavy metals such as Hg are an important component of PM_2.5_ (Yadav and Rajamani 2006). Therefore, it is reasonable for maize whiskers to have a strong retention effect on atmospheric Hg in the natural situation.

In addition, the surface of the maize whiskers is rough and covered with wall folds (Fig. 3). Many studies have shown that rough surface plant structures can more effectively retain atmospheric particulate matter, including Hg (Mo et al. 2015). More importantly, the middle of the maize whiskers is a hollow structure, with both regular catheters and irregular tissue gaps, and SEM measurements (Fig. 3) show that the diameter distribution of these holes ranges from 0.83 to 3.06 μm, which is especially suitable for the adsorption of small substances. It has been shown that maize whiskers can effectively adsorb heavy metal ions such as lead, copper, zinc, and oil substances in wastewater (Petrovic et al. 2016, Petrovic. 2017). In fact, as the female reproductive organs of maize, maize whisker must naturally have the ability to adhere to powdery material such as pollen, and pollen on the stigma germinates into the pollen tube through the hole style to reach the ovary and complete pollination (Duan et al. 2008). Maize whisker must have multiple grading holes as a result of natural selection. Natural cases of corn mainly rely on its surface adsorption of stranded pollutants, and corn powder is bound to further increase the surface area and expose its internal holes, which is further enhanced in this study (Fig. 6).

In addition to physical adsorption, Hg is related to the other retention mechanisms in maize. One is electrostatic adsorption. Maize whiskers contain large amounts of α-reproductive quinones, polysaccharides, and fatty acids, that is, large amounts of hydroxyl, aldehyde, and carboxyl groups (Hasanudin et al. 2012). These negative electric groups can produce electrostatic adsorption with heavy metal ions, which is very conducive to the absorption of heavy metals (Petrovicet al. 2017). The potential is a parameter closely related to electrostatic adsorption, and its numerical size can reflect the interface. In this study, the change in pH value showed an obvious effect on the overall change in zeta potential (Fig. 5). Under acidic conditions, on the one hand, H^+^ will compete with Hg ^2+^ for adsorption; on the other hand, the surface of maize whisker has a large positive charge due to the protonation of hydroxyl and carboxyl groups, which hinders the proximity of metal cations and leads to the low adsorption of Pb, which is consistent with the results of energy spectrum analysis (Additional file 1: Table S1). Therefore, pH is determined by affecting the charge type and amount of charge on the maize whisker surface, thus further affecting the adsorption amount.

The second is chelation. Comparison of the infrared spectra before and after the retention of Hg decreased, and the absorption peaks moved to different degrees (Fig. 4). This is due to the interaction between the functional groups and the metal loaded on the adsorbent, and the intensity and position of the peaks in the infrared spectrum will change (Liu et al. 2021). After Hg treatment, the peak increased from 3451.47 to 3451.89 cm^−1^ due to the stretching vibration of the hydroxyl group, and the peak intensity significantly decreased, probably because of the ion exchange or complexation reaction of Hg^2+^ with the functional groups present in the maize whiskers to form a complex. However, the absorption peaks at 1631 cm^−1^, 1352 cm^−1^, and 1384 cm^−1^ moved to a higher wavenumber, indicating that -OH is involved in the adsorption process. The absorption peaks at 1092 cm^−1^ and 1081 cm^−1^ were shifted, indicating that -O- is involved in the adsorption process. These phenomena suggest that functional groups such as -OH, -COOH, and -O- are involved in the adsorption process.

In addition to physical adsorption, ion exchange may occur during atmospheric Hg retention. The hollow structure in the middle of the maize whiskers enhances its specific surface area to allow more Hg^2+^ diffusion and provides more active sites for stranded Hg (Meng et al. 2018; Zhou et al. 2021). The ion exchange method can be used to remove Hg from wastewater in industry. The principle is that Hg ions in wastewater act as ion exchange agents in ion exchange reactions to remove Hg ions in wastewater (Fang et al. 2018). Chojnacki et al. (2004) treated Hg waste liquid with natural zeolites and found that cations such as Na, K, and Ca in natural zeolites might be replaced by Hg ions. It was speculated that these elements are exchanged with Hg. Davis et al. (2003) used sargasso to absorb Hg ions and found that while sargassum absorbed Hg ions, K and Ca as basic cell wall elements, and some trace elements were replaced in the solution. In this experiment, Hg ions exist on the surface and cross-section of maize whiskers (Fig. 3). Compared with the energy profiles before and after Hg treatment, the peak of Mg decreased after Hg^2+^ adsorption. It is speculated that ion exchange between Hg^2+^ and Mg^2+^ may occur during the retention process.

## Conclusion

This experiment shows that different environmental factors, including initial Hg concentration, maize whisker masses, pH value, and exposure time, have a significant impact on the effect of atmospheric Hg retention with maize whiskers. The comparative analysis of the characteristics of the corn whiskers before and after Hg treatment shows that maize whisker can effectively strand atmospheric Hg, which is associated with a variety of mechanisms, including physical adsorption, electrostatic adsorption, complexation, chelation, and ion exchange. More noteworthy, the maize whisker hole feature has a significant influence on its ability to retain atmospheric Hg, because the correlation analysis shows that maize whiskers have a significant correlation between atmospheric Hg retention and its specific surface area, pore size, medium pore ratio, and micropore ratio. Therefore, this study reveals the important potential value of agricultural waste maize whiskers in the purification of atmospheric heavy metal Hg, which will provide a theoretical basis for its further application to biosorption or the preparation of porous materials.

### Supplementary Information

**Additional file 1: Table S1.** Energy spectrum analysis before and after Hg retention with maize whiskers. **Table S2.** FTIR analysis before and after Hg treatment of maize whiskers. **Fig. S1.** Adsorption-desorption isotherm curves of maize whiskers with different diameters.

## Data Availability

The datasets used and/or analyzed during the current study are available from the corresponding author on reasonable request.
