# Supplementary material for: Retention properties and mechanism of agricultural waste maize whisker on atmospheric mercury
Source: Bioresour Bioprocess. 2023 Sep 29;10(1):67. doi: 10.1186/s40643-023-00683-y (PMC10991902; doi:10.1186/s40643-023-00683-y)
Supplement: Supplementary file 1 — Additional file 1: Table S1. Energy spectrum analysis before and after Hg retention with maize whiskers. Table S2. FTIR analysis before and after Hg treatment of maize whiskers. Fig. S1. Adsorption-desorption isotherm curves of maize whiskers with different diameters. [file 40643_2023_683_MOESM1_ESM.docx]

**Additional file materials**

Retention properties and mechanism of agricultural waste maize whisker on atmospheric mercury

Guiling Zheng^a^, Qianxiu Chen^a^, Feng Zhou^b,*^, Peng Li^a,*^

^a^ School of Resources and Environment, Qingdao Agricultural University, Qingdao 266109, Shandong, China

^b^ School of Food Science, Nanjing Xiaozhuang University, Nanjing 211171, Jiangsu, China

* Corresponding author, E-mail: [pengleep@q](mailto:pengleep@163.com)au.edu.cn

Additional file 1: Table S1. Energy spectrum analysis before and after Hg retention with maize whiskers

| Elements | Before Hg treatment | | After Hg treatment | |
| --- | --- | --- | --- | --- |
|  | Weight percentage (%) | Atom percentage (%) | Weight percentage (%) | Atom percentage (%) |
| C | 55.47±0.41 | 73.14±3.51 | 39.43±4.07 | 62.10±5.16 |
| O | 19.97±6.13 | 22.83±2.24 | 27.53±4.99 | 32.58±6.33 |
| Mg | 0.59±0.03 | 0.46±0.11 | 0.50±0.14 | 0.40±0.07 |
| Si | 3.15±4.47 | 1.84±2.65 | 0.78±0.74 | 0.55±0.49 |
| K | 0.29±0.16 | 0.09±0.03 | 1.05±0.49 | 0.50±0.21 |
| Ca | 0.84±0.25 | 0.29±0.04 | 1.70±0.92 | 0.79±0.46 |
| P | 0.1±0.16 | 0.03±0.04 | 0.33±0.18 | 0.20±0.07 |
| S | 0.36±0.22 | 0.17±0.11 | 0.25±0.14 | 0.15±0.07 |
| Hg | 0 | 0 | 3.65±1.70 | 0.33±0.18 |
| Au | 19.23±13.22 | 1.15±2.05 | 24.78±3.36 | 2.40±0.35 |

Table S2 FTIR analysis before and after Hg treatment of maize whiskers

| Wavelength（cm^-1^） | | Functional group |
| --- | --- | --- |
| Before Hg treatment | After Hg treatment |  |
| 3447.13 | 3439.42 | -OH |
| 2025.85 | 2025.37 | C=H |
| 1507.57 | 1508.06 | N-H/C-N |
| 1384.63 | 1384.15 | -OH |
| 1352.33 | 1353.30 | -OH |
| 1270.86 | 1270.37 | -C-O |


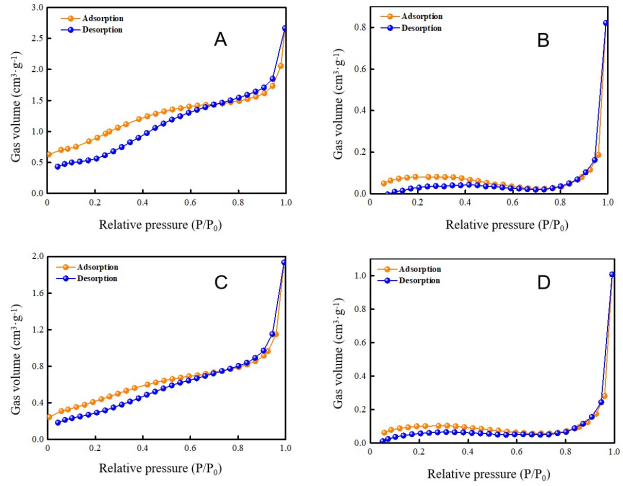


Fig. S1 Adsorption-desorption isotherm curves of maize whiskers with different diameters

Notes: A, maize whiskers with the diameter is 75 μm; B, maize whiskers with the diameter is 100 μm; C, maize whiskers with the diameter is 150 μm; D, maize whiskers with the diameter is 270 μm
